# Algorithmic bias in public health AI: a silent threat to equity in low-resource settings

**DOI:** 10.3389/fpubh.2025.1643180

**Published:** 2025-07-23

**Authors:** Jeena Joseph

**Affiliations:** Department of Computer Applications, Marian College Kuttikkanam Autonomous, Kuttikkanam, Kerala, India

**Keywords:** algorithmic bias, artificial intelligence in public health, health equity, low-resource settings, digital health disparities, inclusive AI

Public health systems have long touted the integration of artificial intelligence (AI) as the game-changing innovation with the potential to transform the delivery of health services, the diagnosis of diseases, and the protection of people in the community. Ranging from sophisticated disease surveillance to computer-aided diagnosis applications, AI is being used in myriad contexts within public health, with the promise to make things more efficient, personalized, and accessible. Underlying all this promise, however, is one much under-investigated concern: the threat posed by algorithmic bias. In contexts where margins for error are thin and exclusion is likely to have high stakes, biased AI systems can widen the existing health gaps instead of bridging them (1). Hidden in code, imperceptible in action, this is an urgent threat to consider.

AI systems are only as effective as the data used to train them and the assumptions under which they are created (2). Many public health AI models, however, draw from data sets in populations which are unrepresentative of those in the low- and middle-income countries (LMICs). The resulting data inequity means the algorithms then do not capture the cultural, linguistic, genetic, or environmental variety in the underserved populations (3). Consequently, health AI systems will systematically underdiagnose, misclassify, or outright ignore patterns in the non-conforming population. This is especially risky in settings where the healthcare infrastructure is already under stress, such as where digital health interventions are viewed as cost-saving measures (4).

Sources of health AI bias are multi-pronged. They begin with the data collection stage, where the majority of data sets come from city hospitals, research centers, or wealthy countries. Such data sets systematically exclude rural patients, ethnic minorities, indigenous people, or socially marginalized groups (2). During the labeling phase, clinical annotations can introduce bias when medical thresholds and definitions are drawn from the dominant population without accounting for cultural or biological variation (5, 6). Algorithmic in its own right, models optimized to be as accurate as possible might disregard considerations of fairness, resulting in consistently disparate performance across subgroups (7, 8). Finally, at the deployment phase, systems can act capriciously if brought into use in settings unlike those in which it has been developed and evaluated (2). Cumulatively, the problems lead to a series of silent reaffirmations of structural disparities.

To more rigorously frame the sources of AI bias, it is important to refer to well-established typologies in algorithmic bias literature. Public health AI typically suffers from historic bias, by which prior injustices—inequities in access to care or discriminatory health policy, say—are embedded within datasets for learning (9, 10). Representation bias is present when samples from urban, wealthy, or connected groups lead to the ignoring of samples from rural, indigenous, or disenfranchised groups (2). In addition, measurement bias is present when health endpoints are approximated with the help of proxy variables—hospital attendance or smartphone usage, say—strikingly different between socioeconomic or even cultural environments (11). These biases are again compounded by aggregation bias, by which models assume homogeneity between heterogeneous groups, and deployment bias, by which tools developed within high-resource environments are simply implemented without modification into low-resource environments (12). Recognition of these forms of bias allows more structured comprehension of how algorithmic harm takes place and where mitigation efforts must be targeted.

Real-world instances of algorithmic bias in public health reinforce the urgency of addressing these typologies. A well-documented case of historic bias is evident in a widely used U.S. healthcare risk prediction algorithm that systematically underestimated the health needs of Black patients by using prior healthcare expenditure as a proxy—unintentionally replicating patterns of historical underutilization of care (13). Representation bias has surfaced in sepsis prediction models developed in high-income settings that showed significantly reduced accuracy among Hispanic patients due to unbalanced training data (14). In the realm of measurement bias, India's digital health initiatives have often relied on smartphone usage for patient engagement, effectively excluding large segments of women, older adult individuals, and rural populations who lack digital access (15). Deployment bias was starkly illustrated during the COVID-19 pandemic through the Aarogya Setu contact tracing app, which failed to reach populations without smartphones, particularly in rural and low-income communities, raising concerns about uneven public health protection (8). These examples underscore that algorithmic bias is not merely theoretical; it has material consequences that can exacerbate the very inequalities public health systems aim to mitigate.

At the heart of algorithmic bias lies the issue of structural data exclusion. Many AI systems draw their inputs from data sources that systematically omit rural populations, marginalized castes, indigenous groups, or those without digital access. This exclusion is not accidental—it is a byproduct of how data is collected, labeled, and interpreted (16). Clinical annotations are often standardized using thresholds derived from dominant populations, ignoring genetic, environmental, or cultural differences that influence health outcomes (17). When these biased models are used in low-resource contexts, they risk making inaccurate diagnoses or failing to detect public health emergencies altogether (18). This is not a technical shortcoming but a structural flaw, one that reflects who is seen, heard, and counted in the design of digital health tools.

These effects of algorithmic prejudice in public health do not remain theoretical. They appear as material harm. In Brazil, AI models trained with city-level data did not capture rural disease epidemics from environmental and socio-economic features missing from the training data (19). In India, mobile applications using smartphone availability for self-reporting or tele-consultation exclude significant parts of the population who have no familiarity with digital technologies or simply no means to own or access mobile phones (20, 21). Such exclusion is neither a technical error nor simply an oversight; it is the further extension of historic inequalities now replicated through ostensibly neutral technology. The illusory assumption that technology has intrinsic objectivity obscures the need for contextual intelligence in the crafting and deployment of systems.

A central risk in today's AI-in-health interventions is the myth of neutrality. It is assumed frequently that AI systems lack biases inherent in human beings due to their use of data and statistically grounded reasoning. AI models, however, are created, trained, and evaluated by people, and cannot help but reflect the developers' assumptions, tastes, and blind spots. This is particularly problematic where AI development is concentrated in the Global North, and deployment in LMICs has radically dissimilar social and health contexts (22). The outcome is one digital colonialism where technologies created in one context get transferred to another with little or no adaptation or local contribution.

This myth of neutrality obscures the fact that AI systems are human artifacts—shaped by the data they are trained on, the objectives they are given, and the assumptions of their developers. Public health AI often reflects the institutional biases of the healthcare systems and research ecosystems from which it originates (11). For instance, the development of AI tools predominantly in high-income countries leads to the encoding of social, cultural, and biological norms that are misaligned with the realities of low-resource settings (23). When these systems are deployed in vastly different environments, they bring with them embedded assumptions that do not travel well. As a result, the notion that AI offers a ‘scientific' or ‘impartial' solution to global health problems becomes not only misleading but dangerous—especially when it prevents critical reflection or accountability for systemic failures in underrepresented communities (24).

Public health systems in low-resource settings tend to be poorly equipped to detect or respond to the impacts of biased AI. They lack effective regulatory frameworks to govern AI, have weak or unenforced data protection laws, or lack the technical skills to conduct audits of AI systems (25). This is a perfect storm in which suboptimal algorithms can be injected into national health policy with inadequate oversight. Digital exclusion further exacerbates these dynamics. Underprivileged populations who already have inadequate access to healthcare services are also likely to be marginalized from digital interventions, further entrenching inequalities (23).

Tackling algorithmic bias in public health requires more than technical interventions—it demands a reengineering of values into systems (26). Equity cannot be retrofitted; it must be a foundational design principle. This means ensuring that development teams are multidisciplinary and inclusive of voices from the Global South, marginalized communities, and local health ecosystems (27). Participatory design, where affected populations co-create and critique AI tools, should be standard practice rather than an afterthought (28). Moreover, the use of fairness audits, synthetic data for underrepresented cases, and multilingual NLP models can help mitigate systemic blind spots (29). Countries like India, with their complex demographic and linguistic diversity, offer both cautionary tales and promising models. If India pioneers equity-focused AI governance, it could serve as a replicable framework for other low- and middle-income countries facing similar challenges. To meet these challenges, we need a paradigm shift in the conceptualization, development, and deployment of AI for public health. More than technical sophistication or predictive precision, developers and policymakers need to support an equity-focused orientation. This work would start with inclusive data practices with the express goal to capture the full diversity of the population. Strategies in data collection need to be redesigned to encompass rural areas, under-represented languages, and marginalized groups (30). Local health workers as well as community-based organizations can be critical allies in the creation of representative as well as relevant.

Bias reduction should also be integrated into the development and assessment of AI systems. AI tools should be subject to fairness audits in front of deployment to examine their accuracy against different demographic and socio-economic groups (31). Imbalances in the accuracy of predictions, false positives, and false negatives should be recorded and rectified. Crucially, such evaluations should not be looked at as singular processes but as continuing routines in the life cycle of the AI system (26). Transparency and explainability should be valued as well. Public health officials as well as community stakeholders should have insight into the workings of these systems, the data used, and the assumptions.

Health technologists need to collaborate with social scientists, public health practitioners, ethicists, and impacted communities to ensure AI systems remain contextually situated. Such collaboration should not only occur in the development stage but also in deployment, monitoring, and evaluation. Participatory methods, in which the individuals at the heart of the issue participate in defining the tools impacting them, can bridge the disconnect between technical innovation and everyday experience (32).

One new path to enhancing equity in public health AI is to utilize synthetic data generation to bridge the gaps in under-representative populations—as long as it is carried out ethically (33). Generative models such as GANs can, for instance, mimic data from rare diseases or rural settings where authentic data is in short supply. This cannot entirely supplant genuine field data, but it can supplement it at early stages to circumvent algorithmic blind spots. Also, incorporating local languages and indigenous knowledge into NLP-based health applications has the potential to expand coverage in multilingual cultures (34). Decentralized AI architectures, in which models get trained locally using varied datasets in place of central data repositories, have as-yet untapped potential. Such methods could limit prejudice and amplify cultural emphasis.

India is an interesting case study in the intersection of AI, public health, and equity. With its demographics, geographic variation, and hybrid public-private healthcare delivery system, India has adopted AI at breakneck velocity in applications from disease surveillance to diagnosis to telemedicine (35). The velocity has, to date, not been paired with a strong framework to determine the threat of algorithmic bias. The Aarogya Setu app used in the COVID-19 response to track contacts serves to highlight the strength as well as the limitations of digital public health technologies (36). It reached the millions, but only those with smartphones, with severe privacy as well as data governance worries. AI models to predict diabetes as well as cardiovascular risk have had promise, but whether their findings can generalize to the varied subgroups in India is uncertain given the variation in regions in terms of dietary intake, lifestyle, as well as availability of care.

India's experience highlights the necessity to develop country-specific strategies for AI in consideration of socio-cultural, linguistic, and infrastructural diversity (35). Developing AI systems in India with ethics and awareness about biases needs technological investment as well as in local research, grassroots engagement, and policy reform. If India is successful in developing an inclusive AI environment, it can be replicated in similar challenges in other LMICs.

No single stakeholder is responsible for addressing algorithmic bias in public health AI. It is the collective responsibility of governments, developers, funders, researchers, and communities (37). International reformers, international funders, and global health institutions need to make bias assessments and equity measures mandatory as part of project proposals and program evaluations (16). Tech companies and research institutions need to put open-source models and data sets with global diversity as their priority. International regulators need to frame explicit guidelines for the deployment of ethical AI with mechanisms to ensure community redress and accountability (38).

In the end, the goal is not to eliminate AI but to rethink it as an inclusionary technology, rather than an exclusion technology. This means designing systems which do not simply reproduce what is effective in privileged situations but also develop in accordance with the needs of those who have long been unserved. It means designing feedback mechanisms in which users can challenge, critique, and shape AI systems. It means committing to the value that equity needs to be engineered—and not just assumed.

In the years to come, artificial intelligence will further leave its mark in world health. As it does so, the hidden biases embedded in algorithms will determine who is diagnosed, who is treated, and who is left behind. The stakes could hardly be higher. Without conscious effort to detect, minimize, and block algorithmic bias, we risk automating injustice and entrenching inequality in the very technologies we hope to enhance. The time to act is now, before such biases harden into the default reasoning of digital health.

By making fairness, transparency, and inclusivity the foundation in the development and deployment of public health AI, we can turn the potential threat into an empowering force toward health equity. It will take more than good intentions to do it, however; it will take critical analysis, system reform, as well as the willingness to break the seductive myth about neutrality.
